# Association between triglyceride-glucose index and estimated 10-year risk of cardiovascular disease in Nigerian non-diabetic hypertensives: A cross-sectional study

**DOI:** 10.1371/journal.pgph.0004760

**Published:** 2025-07-01

**Authors:** Casmir E. Amadi, Ugochi C. Okorafor, Chiamaka I. Okorafor, Micah N. Okwah, Precious O. Akanbi, Onyinyechi V. Okam, Christie I. Udenze, Amam C. Mbakwem, Jayne N. Ajuluchukwu

**Affiliations:** 1 Department of Medicine, College of Medicine, University of Lagos, Lagos, Nigeria; 2 Acute Specialty Medicine, King’s College Hospital, London, United Kingdom; 3 Festac Primary Health Centre, Amuwo-Odofin, Lagos, Nigeria; 4 Lagos University Teaching Hospital, Lagos, Nigeria; 5 Babcock University Teaching Hospital, Ilishan-Remo, Ogun State, Nigeria; 6 Department of Chemical Pathology, College of Medicine, University of Lagos, Lagos, Nigeria; Mercer University School of Medicine, UNITED STATES OF AMERICA

## Abstract

The Triglyceride Glucose (TyG) index is a new and sensitive marker of Insulin Resistance (IR). Its relationship with a 10-year cardiovascular risk (CVD) in hypertensives in Nigeria is currently unknown. This was a cross-sectional study involving 1,430 adult Nigerians with hypertension. The TyG index of subjects was divided into quartiles (Q1-Q4), and its association with 10-year CVD risk using Framingham 10-year Cardiovascular Risk Score was explored. The mean age of the study population was 55.8 ± 13.9 years with a male preponderance of 54.1%. The mean TyG index was 8.72 ± 0.6 with a significantly higher value in males (8.77 ± 0.6 vs 8.67 ± 0.5; p = 0.001). This trend was consistent across all age groups. The prevalence of elevated TyG index was 51.9% and 44.7% in males and females respectively. Using ROC the cut-offs that predicted moderate to high CVD risk were 8.85 (0.69(0.57-0.80; 95% CI; p = 0.001) and 8.74 (0.688 (0.59-0.78; 95% CI; p = 0.001) for males and females respectively. After adjustment for well-known risk factors, an elevated TyG index was associated with an increased 10-year CVD risk in females only. The TyG index is associated with an increased 10-year cardiovascular risk especially in females and a value of at least 8.7 is reasonably accurate to predict a 10-year CVD risk in Nigerians with hypertension.

## Introduction

Insulin resistance (IR) is a pathological state characterized by the attenuated response of insulin-sensitive tissues (skeletal and cardiac muscle, adipose tissue, and liver) to insulin signaling [1,2]. This leads to metabolic remodelling characterised by the impairment of glucose uptake by the cells as their primary energy substrate. The downstream effects of this impairment include clinical conditions (chronic hyperglycemia, obesity, hypertension and dyslipidemia) and pathological substrates (low-grade inflammation, endothelial dysfunction and atherosclerosis), all of which are associated with the promotion of CVD either as risk factors or pathogenetic substrates [3,4]. Atherosclerotic cardiovascular disease (ASCVD) is the number one cause of preventable and premature morbidity and mortality globally, accounting for about 18 million deaths in 2019 [5]. IR is a major driver of ASCVD because of its underpinning with the traditional risk factors for CVD (hypertension, obesity, type 2 diabetes and dyslipidemia) and chronic inflammation [6,7].

The gold standard for measuring IR is the euglycemic–hyperinsulinemic clamp. However, this method is limited by its technicalities and cost (money and time resources) in routine clinical practice [8]. The Homeostatic Model Assessment for Insulin Resistance (HOMA-IR) is a surrogate marker of IR. Still, its use is also limited by the cost of measuring insulin, and it is also unreliable in individuals receiving insulin and those without functioning β-cells [8,9]. The triglyceride-glucose index (TyG index), introduced in 2008, is a composite of fasting triglyceride and blood glucose. It is a simple, reliable, and low-cost (with few constraints on time and money) surrogate of IR due to its high sensitivity and specificity. Given these, it comes in handy in clinical and epidemiological settings and is said to be as accurate as (perhaps more precise than) the HOMA-IR in evaluating insulin resistance [10,11]. TyG index is the logarithmic product of fasting triglyceride and fasting blood glucose (easily measurable parameters in clinical settings). It is associated with the risk of coronary artery disease, stroke, heart failure, and obesity-related diseases, type 2 diabetes, all-cause mortality in large homogeneous populations [12–15].

Hypertension is one of the most critical risk factors for ASCVD and shares a bi-directional relationship with IR [16]. In Nigeria, the prevalence of hypertension is about 31% [17]. It is the premier precursor of ASCVD, especially stroke. ASCVD is caused by a constellation of frequently co-occurring risk factors which have a multiplicative effect on overall ASCVD risk assessed by the Framingham Risk Score (FRS) and other risk estimators [18]. Preventive cardiology aims to prevent the first ever CVD (primary prevention) through opportunistic screening for potentially sanctionable risk factors, risk stratifying individuals and mitigating this risk.

In Nigeria, the prevalence of IR in individuals with hypertension is as high as 31.4%, putting them at a very high risk for ASCVD (from the hypertension itself and the IR [19]. These individuals are meant to be screened for dyslipidemia and type 2 diabetes in line with both National and International guidelines on the management of hypertension [20,21]. This practice yields two parameters (fasting triglyceride and glucose) that can be used to assess IR using the TyG index. Despite the high prevalence of hypertension and insulin resistance in hypertensives in Nigeria, the association between IR and CVD risk using the TyG index remains under-explored. This study was designed to evaluate the predictive value of the TyG index for 10-year CVD risk using the FRS and determine an optimal TyG index cut-off for moderate to high CVD risk in a population of adult Nigerians with hypertension.

## Methods

### Study population

This cross-sectional retrospective study reviewed the hospital records of adult hypertensives who presented at a Cardiac Centre in Lagos, Nigeria, over 15 years (2009–2024). The hospital records of 1,430 hypertensives with complete data were retrieved for analysis between April 1^st^ and December 19^th^ 2024. All individuals with hypertension presenting for the first time at the Cardiac Center are extensively evaluated by the cardiologists and trained nurses usually extensively evaluate all hypertensives in line with both the National and International guidelines for the management of hypertension. This evaluation includes their socio-demographic characteristics, anthropometric indices, lifestyle risk factors, medical history and thorough physical examination. After clinical evaluation, these patients are requested to undergo laboratory evaluation, which includes fasting lipid profile, fasting plasma glucose, serum creatinine and serum uric acid, among others. For this study, hypertensives with diabetes, stroke, heart failure, atrial fibrillation, heart failure, Stage IV and V chronic kidney disease and coronary heart diseases were excluded.

### Ethics approval

Since the data of the subjects were completely de-identified and anonymized the Health Research Ethics Committee of the Lagos University Teaching Hospital, Idi-Araba, Lagos, Nigeria, waived the requirement for informed consent (with identification number ADM/DSCST/HREC/APP/6494).

### Data collection/measurements

Anonymised and de-identified socio-demographic details, anthropometric measurements (Body Mass Index and Waist Circumference), medical histories and laboratory results were retrieved from the records and transferred into an electronic case report form. TyG index was derived as Ln (Fasting triglyceride (mg/dl) x fasting plasma glucose (mg/dl)/2 [17]. TyG index was divided into quartiles: Q1 (<8.37), Q2 (8.37-8.70), Q3 (8.71-8.94) and Q4 (>8.94) respectively. The cuff off for elevated TyG index was taken to be 4.74, 8.8 and 8.73 in the total population, males and females respectively. These cut-off points have been shown to predict CVD risk and metabolic syndrome in the metabolically healthy population [22].

The Framingham Risk Score was used to estimate the subjects’ risk of developing cardiovascular disease in 10 years using the age and sex of the patient, history of smoking, hypertension (and on anti-hypertensive medications) and diabetes, along with the values for systolic blood pressure, total and high-density lipoprotein (HDL) cholesterol using an online calculator. The scores were categorized into low, moderate and high risk (< 10%, 10% to <20% and ≥20%, respectively).

### Statistical analysis

Continuous variables were expressed as mean ± standard deviation (SD) for normally distributed data or median and interquartile range when skewed, while categorical variables were expressed as percentages. Comparisons between groups were made with the Student’s t-test and χ^2^ for continuous and categorical data, respectively. Prespecified analyses included linear regression which was used to explore the relationship between TyG index and 10-year CVD risk in males and females and multivariable logistic regression, adjusted in three models: unadjusted (Model 1), adjusted for age and gender (Model 2), and fully adjusted for cardiovascular risk factors (Model 3). Receiver Operating Characteristic (ROC) analysis was prespecified to determine the optimal TyG index cut-off for predicting moderate-to-high CVD risk, with area under the curve (AUC) values reported for model performance. Exploratory analyses included subgroup analyses by sex (male vs. female) and age groups (<60 years vs. ≥ 60 years) to determine whether associations varied across demographics. All statistical analyses were performed using SPSS version 28.0 software (SPSS Inc, Chicago, IL), and a p < 0.05 was considered statistically significant. Charts were used for data presentation where appropriate.

## Results

### General characteristics of the study population

The study included 1,430 subjects, with a male preponderance (54.1%) and a mean age of 55.8 years (Table 1). The majority (70%) were between 40 and 70 years old. Smoking and alcohol use were reported in 4.6% and 26.4% of the subjects, respectively. The mean TyG index was 8.7, significantly higher in males (p < 0.001). Males also had a higher 10-year CVD risk than females (p < 0.001).

**Table 1:** Socio-demographic and clinical characteristics of participants.

	Overall	Male	Female	p-value
Age group (Years)				
≤40	200 (13.6)	100 (12.9)	100 (14.4)	0.021*
41–50	382 (26.0)	222 (28.7)	160 (23.0)	
51–60	352 (23.9)	196 (25.3)	156 (22.4)	
61–70	300 (20.4)	144 (18.6)	156 (22.4)	
>70	236 (16.1)	112 (14.5)	124 (17.8)	
Mean±SD	55.84 ± 13.9	55.11 ± 13.8	56.66 ± 13.9	0.032*
Alcohol consumption				
Yes	388 (26.4)	308 (39.8)	80 (11.5)	<0.001*
No	1082 (73.6)	466 (60.2)	616 (88.5)	
Smoking				
Yes	68 (4.6)	64 (8.3)	4 (0.6)	<0.001*
No	1402 (95.4)	710 (91.7)	692 (99.4)	
Weight (Mean±SD)	89.2 ± 19.0	90.57 ± 17.5	87.65 ± 20.5	0.003*
Height (Mean±SD)	1.68 ± 0.1	1.73 ± 0.1	1.62 ± 0.1	<0.001*
BMI (Mean±SD)	31.73 ± 6.6	30.28 ± 5.5	33.35 ± 7.2	<0.001*
WC (Mean±SD)	105.48 ± 15.9	104.37 ± 16.7	106.70 ± 14.8	0.005*
SBP (Mean±SD)	154.81 ± 24.3	153.74 ± 24.9	155.99 ± 23.6	0.076
DBP (Mean±SD)	89.31 ± 14.5	89.79 ± 14.4	88.78 ± 14.5	0.179
Urea (Median (QR))	33.63 (26.7–42.6)	35.6 (28.1–44.6)	31.4 (25.1–39.3)	<0.001*
Creatinine (Median (QR))	1.10 (0.9–1.3)	1.17 (1.0–1.4)	1.00 (0.9–1.2)	<0.001*
Uric acid (Mean±SD)	7.25 ± 2.3	7.77 ± 2.5	6.69 ± 1.9	<0.001*
LDL-c (Median (QR))	122.3 (97.0–146.6)	118.0 (90.8–143.2)	126.6 (102.8–152.7)	<0.001*
HDL-c Median (QR))	50.2 (42.3–59.7)	47.9 (40.6–58.2)	52.7 (44.6–60.9)	<0.001*
Tc (Median (QR))	196.6 (171.5–227.6)	193.7 (165.6–223.0)	200.9 (176.0–231.7)	<0.001*
VLDL (Median (QR))	22.3 (16.8–30.1)	23.2 (17.3–32.7)	21.6 (16.5–27.6)	<0.001*
TG (Median (QR))	119.1 (88.9–155.6)	123.7 (93.2–170.5)	112.7 (84.5–145.2)	<0.001*
FBG (Mean±SD)	108.9 ± 38.3	108.02 ± 39.7	109.85 ± 36	0.360
HBA1C (Mean±SD)	6.3 ± 1.4	6.29 ± 1.5	6.29 ± 1.3	1.000
eGFR (Median (QR))	65.9 (52.4–81.4)	71.5 (55.4–84.7)	61.3 (49.2–74.7)	<0.001*
10 years CVD risk (Median (QR))	6.0 (2.0–14.0)	10.0 (4.0–20.0)	4.0 (1.0–8.0)	<0.001*
Triglyceride glucose index	8.72 ± 0.6	8.77 ± 0.6	8.67 ± 0.5	0.001*

Legend: BMI = Body Mass Index; WC = Waist circumference; SBP = Systolic Blood Pressure; DBP = Diastolic Blood Pressure; LDL-c = Low Density Lipoprotein cholesterol; HDL-c = High Density Lipoprotein Cholesterol, TG = Triglyceride; FBG = Fasting Blood Glucose; VLDL = Very low density Lipoprotein; eGFR = Estimated glomerular Filtration Rate.

**Fig 1:**
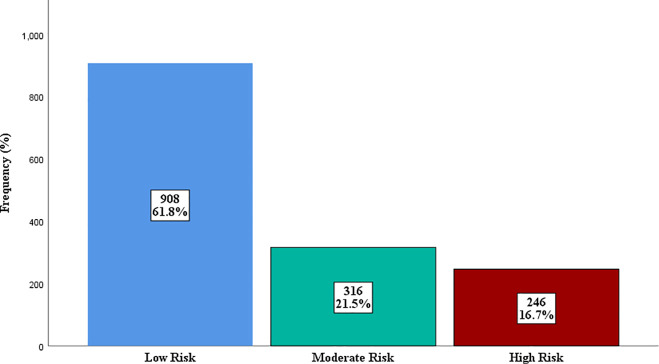
Distribution of Framingham Risk Score categories among the subjects.

The distribution of Framingham Risk Score categories is shown in Fig 1, with approximately one-third of participants classified as having moderate or high CVD risk.

### Prevalence of elevated triglyceride glucose index

**Fig 2:**
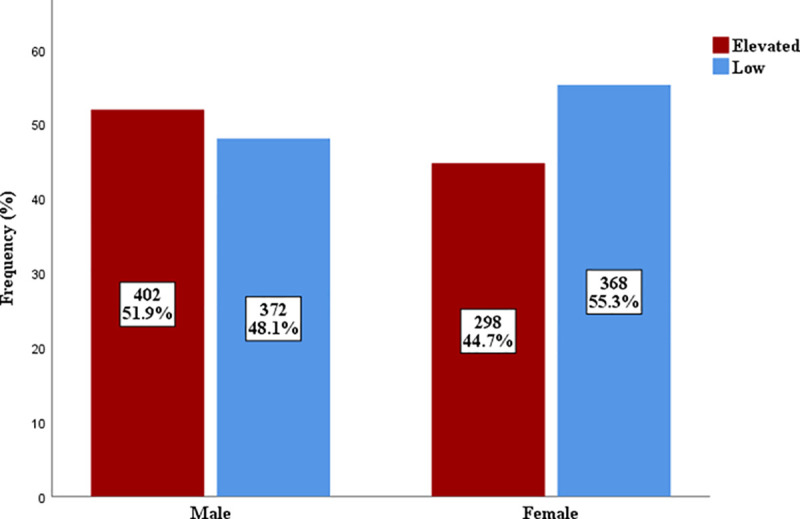
Prevalence of elevated TyG index in males and females.

Using the TyG index cut-offs of 4.74, 8.8 and 8.73 for the general population, males and females respectively, 51.9% and 44.7% of male and female subjects had elevated TyG index respectively (Fig 2).

**Fig 3:**
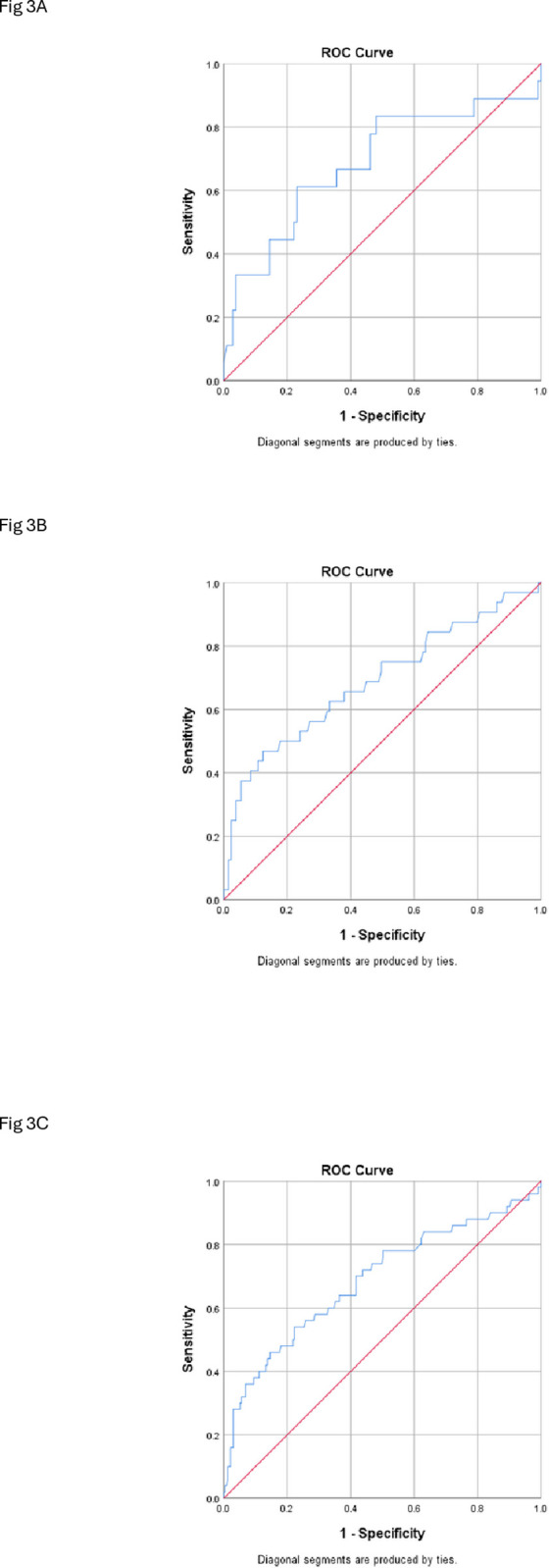
A. receiver operating characteristics of TyG index in predicting 10-year CVD risk. B. Receiver Operating Characteristics of TyG index in predicting 10-year CVD risk. C. Receiver Operating Characteristics of TyG index in predicting 10-year CVD risk.

The lowest TyG index was was 5.7 implying that no subject had normal TyG index. Fig 3 shows a family of ROC curves of TyG index and its ability to predict 10-year CVD risk while Table 2 shows the AUC. In males a cut-off of 8.8 with a sensitivity and specificity of 68.2% and 69.9% respectively predicted moderate to high 10-year CVD risk(95% CI; p < 0.001). Similar trend was observed in females.

**Table 2:** The predictability of TyG index of 10-year CVD risk using RO.

	Cut off	AUC (95% CI)	Sensitivity	Specificity	PPV	NPV	p-value
Overall	8.73	0.69 (0.60–0.78)	0.73	0.67	0.87	0.81	<0.001*
Male	8.85	0.69 (0.58–0.80)	0.68	0.70	0.86	0.83	<0.001*
Female	8.68	0.69 (0.53–0.84)	0.69	0.68	0.81	0.88	<0.001*

AUC = Area under curve

### Patterns of triglyceride glucose index in the subjects

The TyG index was consistently higher in males across all age groups (Table 3). Mean TyG index was significantly higher in males; p = 0.001 and those below age 40 years and between ages 51 and 60 years.

**Table 3:** Age and sex distribution of triglyceride glucose index among participants.

	Male			Female			p-value
	n	Mean±SD	95%CI	n	Mean±SD	95%CI	
Total	774	8.77 ± 0.61		696	8.67 ± 0.5		0.001*
Age group							
≤40	100	8.86 ± 0.58	8.75-8.97	100	8.58 ± 0.48	8.76-8.94	<0.001*
41–50	222	8.72 ± 0.70	8.63-8.81	160	8.69 ± 0.46	8.62-8.76	0.544
51–60	196	8.79 ± 0.50	8.72-8.86	156	8.64 ± 0.55	8.55-8.73	0.007*
61–70	144	8.80 ± 0.66	8.69-8.91	156	8.73 ± 0.46	8.66-8.80	0.301
>70	112	8.71 ± 0.57	8.60-8.82	124	8.70 ± 0.57	8.60-8.80	0.854

When TyG index was stratified into quartiles, systolic blood pressure (SBP), diastolic blood pressure (DBP), fasting blood glucose (FBG), total cholesterol (TC), triglyceride (TG), low-density lipoprotein cholesterol (LDL-c), creatinine, uric acid, and HbA1c increased across the quartiles, whereas high-denisty lipoprotein-cholesterol (HDL-c) and estimated glomerular filtration rate (eGFR) were lower in higher quartiles (Q3 and Q4) (Table 4).

**Table 4:** Socio-demographic and clinical parameters of subjects and distribution of TyG index by quartiles.

TyG index	Q1(5.44-8.37)	Q2(8.37-8.70)	Q3(8.70-9.05)	Q3(9.05-10.86)	p-value
Age	56.93 ± 14.2	55.73 ± 14.1	55.34 ± 13.5	56.37 ± 13.6	0.069
Sex					
Male	167 (45.5)	185 (50.3)	192 (52.3)	230 (62.5)	<0.001*
Female	200 (54.5)	183 (49.7)	175 (47.7)	138 (37.5)	
Alcohol consumption					
Yes	84 (22.9)	88 (23.9)	102 (27.8)	114 (31.0)	0.049*
No	283 (77.1)	280 (76.1)	265 (72.2)	254 (69.0)	
Smoking					
Yes	10 (2.7)	14 (3.8)	18 (4.9)	26 (7.1)	0.035*
No	357 (97.3)	354 (96.2)	349 (95.1)	342 (92.9)	
Weight	87.54 ± 17.1	88.37 ± 19.9	90.88 ± 20.2	89.95 ± 18.7	0.075
BMI	31.56 ± 6.0	31.63 ± 7.3	32.11 ± 6.6	31.63 ± 6.3	0.646
WC	103.40 ± 14.8	106.47 ± 18.6	105.86 ± 16.1	106.21 ± 13.3	0.033*
SBP	153.48 ± 22.2	156.96 ± 26.6	156.81 ± 22.1	157.96 ± 25.4	<0.001*
DBP	87.44 ± 13.5	90.39 ± 15.0	88.89 ± 13.9	90.52 ± 15.1	0.011*
FBS	97.66 ± 18.1	98.49 ± 19.4	105.68 ± 20.7	143.64 ± 26.4	<0.001*
HBA1C	6.03 ± 1.3	5.98 ± 1.2	6.33 ± 1.4	6.83 ± 1.7	<0.001*
Urea	34.75 ± 9.3	35.53 ± .6	34.50 ± 8.8	44.87 ± 9.8	<0.001*
Creatinine	1.12 ± 0.3	1.13 ± 0.3	1.12 ± 0.3	1.50 ± 1.1	<0.001*
Uric acid	7.06 ± 1.1	6.97 ± 1.9	7.08 ± 1.9	7.92 ± 1.9	<0.001*
LDL-c	120.92 ± 21.3	130.82 ± 19.7	123.09 ± 21.3	120.90 ± 20.9	0.003*
HDL-c	50.03 ± 12.2	53.93 ± 11.9	49.66 ± 13.1	42.30 ± 13.8	<0.001*
TC	186.27 ± 36.8	186.27 ± 36.7	204.90 ± 32.1	198.90 ± 34.5	<0.001*
VLDL	15.51 ± 3.4	20.78 ± 4.6	26.52 ± 6.2	31.67 ± 5.4	<0.001*
TG	78.14 ± 19.9	108.98 ± 22.6	140.26 ± 32.4	197.71 ± 34.2	<0.001*
eGFR	70.44 ± 21.6	67.46 ± 19.8	70.02 ± 20.1	60.35 ± 24.2	<0.001*

Legend: BMI = Body Mass Index; WC = Waist circumference; SBP = Systolic Blood Pressure; DBP = Diastolic Blood Pressure; LDL-c = Low Density Lipoprotein cholesterol; HDL-c = High Density Lipoprotein Cholesterol, TG = Triglyceride; FBG = Fasting Blood Glucose; VLDL = Very low density Lipoprotein; eGFR = Estimated glomerular Filtration Rate.

**Fig 4:**
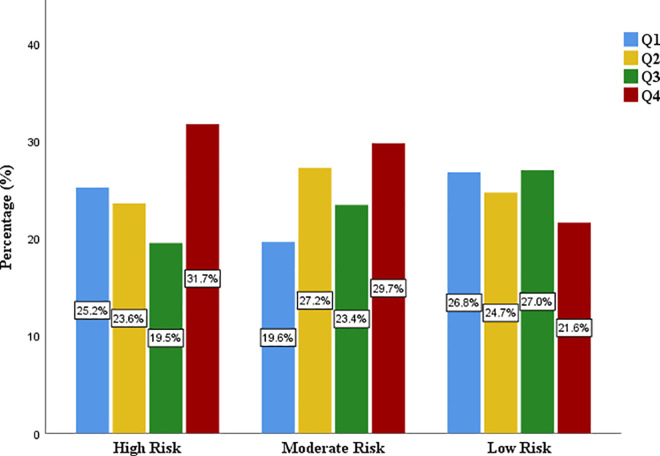
Distribution of 10-year CVD risk by quratiles of TyG index.

With respect to estimated 10-year CVD risk the proportion of the subjects with either moderate or high risk was highest in the Q4 as shown in Fig 4.

### Age and sex-adjusted logistic regression analysis between CVD risk and quartiles of TyG index

Regression analysis was used to explore the possible association of age and sex, the risk of CVD with the quartiles of TyG index. Table 5 shows the odd ratios of the association of age, sex, CVD risk and quartiles of TyG index. In the unadjusted model, the highest risk of 10-year CVD was seen in the fourth quartile of the TyG index. In males and females only the Q3 and Q4 were associated with increased odds for CVD risk risk: 1.86{1.05-2.3; 95% CI; p = 0.011 and 2.54(0.77-1.67; 95% CI; p < 0.001} for males and 1.42{1.21-2.84; 95% CI; p = 0.014 and 2.18(1.37-3.47; 95% CI; p < 0.001} for females. For age Q3 and Q4 were associated with increased 10-year CVD only in those above age 60; 0.35(0.17-0.71; 95% CI; p = 0.004 and 2.33(1.23-4.39; 95% CI; p = 0.009 respectively.

**Table 5:** Age and Sex adjusted logistic regression of 10-year CVD risk and triglyceride glucose index among participants.

	Odd ratio	95% CI	p-value
**TyG index (Quartile)**			
Quartile 1 (<8.38)	1		
Quartile 2 (8.38–8.70)	1.18	0.86-1.62	0.302
Quartile 3 (8.71–8.94)	0.73	0.51-1.04	0.081
Quartile 4 (>8.94)	1.45	1.09-1.97	0.012*
**Male**			
**TyG index (Quartile)**			
Quartile 1 (<8.38)	1		
Quartile 2 (8.38–8.70)	1.59	0.82-1.92	0.178
Quartile 3 (8.71–8.94)	1.86	1.05-2.34	0.011*
Quartile 4 (>8.94)	2.54	0.77-1.67	0.001*
**Female**			
**TyG index (Quartile)**			
Quartile 1 (<8.38)	1		
Quartile 2 (8.38–8.70)	1.26	0.66-1.79	0.745
Quartile 3 (8.71–8.94)	1.42	1.21-2.84	0.014*
Quartile 4 (>8.94)	2.18	1.37-3.47	0.001*
**Age ≤ 60**			
**TyG index (Quartile)**			
Quartile 1 (<8.38)	1		
Quartile 2 (8.38–8.70)	1.47	0.82-2.63	0.196
Quartile 3 (8.71–8.94)	0.93	0.49-1.74	0.816
Quartile 4 (>8.94)	1.64	0.96-2.80	0.068
**Age > 60**			
**TyG index (Quartile)**			
Quartile 1 (<8.38)	1		
Quartile 2 (8.38–8.70)	1.59	0.82-3.08	0.172
Quartile 3 (8.71–8.94)	0.35	0.17-0.71	0.004*
Quartile 4 (>8.94)	2.33	1.23-4.39	0.009*

Adjusted for Age, BMI, WC, sex, alcohol consumption, smoking

### Association between TyG index and 10-year CVD risk

Table 6 shows further exploratory analyses using multivariate regression to seek for an association between TyG index and 10-year CVD risk. Model 1 was unadjusted; Model 2 was adjusted for age and gender, while Model 3 adjusted for age, gender, BMI, SBP, DBP, HDL-c, LDL-c, TC, and TG. Our results show that the TyG index was associated with an increased 10-year risk of CVD in females only. Table 7 shows the logistic regression analysis of TyG index and 10-year risk of of CVD by quartiles. Compared to Q1, the 10 year risk of CVD was higher in Q3 and Q4 1.42(1.21-2.84); p = 0.014; and 2.18(1.37-3.48); p = 0.001 respectively. In model 2 the CVD risk was higher in Q4 only; 1.45(1.09-1.97); p = 0.012.

**Table 6:** Linear regression analysis of TyG index and 10-year risk of CVD risk in males and females.

	Model 1	Model 2	Model 3
	B (95% CI) p-value	B (95% CI) p-value	B (95% CI) p-value
Male	1.13(0.90-1.43) p = 0.286	1.08(0.93-1.37) P = 0.464	1.39(0.94-1.09) p = 0.854
Female	1.67 (1.18-2.35) p = 0.004	1.48(1.23-1.39) p = 0.010*	1.38(1.04-2.37) p = 0.032*

Model 1 adjusts for none; Model 2 adjusts for age and sex; Model 3 adjusts for age, sex, BMI, WC, SBP, DBP, HDL-c, LDL-c, TC, TG; CI = Confidence interval.

**Table 7:** Logistic Regression analysis of TyG index and 10-year risk of CVD.

	Model 1	Model 2	Model 3
	OR (95% CI) p-value	OR (95% CI) p-value	OR (95% CI) p-value
Q2 vs Q1	1.09(0.66-1.79); p = 0.745	1.18(0.86-1.62);p = 0.302	1.47(0.82-2.63)p = 0.196
Q3 vs Q1	1.419(1.21-2.84); p = 0.014*	1.23(0.91-1.64);p = 0.081	0.93(0.49-1.74)p = 0.816
Q4 vs Q1	2.180(1.37-3.47); p = 0.001*	1.45(1.09-1.97); p = 0.012*	1.64(0.96-2.80)p = 0.068

Model 1 adjusts for none; Model 2 adjusts for age and sex; Model 3 adjusts for age, sex, BMI, WC, SBP, DBP, HDL-c, LDL-c, TC, TG; CI = Confidence interval

## Discussion

To our knowledge, this is the first study in Nigeria to examine the association between insulin resistance (IR), assessed using the Triglyceride-Glucose (TyG) index, and estimated 10-year cardiovascular disease (CVD) risk. Our findings demonstrate a positive but non-linear association between the TyG index and 10-year CVD risk, with subjects in the higher TyG index quartiles exhibiting a greater risk. This association remained statistically significant after adjusting for traditional atherosclerotic cardiovascular disease (ASCVD) risk factors, including age, sex, and blood pressure. However, this observational study does not establish causality, and the results should be interpreted within this context. Our study was also able to determine the TyG index index cut-off that reasonably predicted moderate to high 10-year CVD risk using ROC curves. These results suggest that the TyG index may be a valuable tool for cardiovascular risk stratification in Nigerian hypertensives, potentially complementing existing risk assessment models.

In the existing literature, the TyG index has been strongly associated with ASCVD and other CVDs, independent of confounding factors. Studies have consistently reported a non-linear relationship, where individuals in the higher TyG index quartiles experience greater CVD risk, a trend that aligns with our findings [23–27]. The association between the TyG index and CVD risk has been extensively studied in Asian populations, with multiple studies demonstrating its predictive value. Research from Korea has shown that the TyG index may serve as a useful marker for CVD risk stratification in individuals older than 40 years and young adults aged 20–39 years [28,29]. Similarly, Guo et al. reported that the TyG index was independently associated with arterial stiffness and 10-year CVD risk, using the Framingham Risk Score (FRS) in a cohort of apparently healthy Chinese individuals [30]. The Kailuan study in Northern China also documented a positive association between the TyG index and CVD risk [31]. Moreover, studies indicate that higher TyG quartiles correlate with worse CVD outcomes [32–34]. The biological mechanisms linking the TyG index to increased CVD risk are well established. IR is a key driver of low-grade inflammation, oxidative stress, endothelial dysfunction, and coagulation abnormalities, all contributing to CVD pathogenesis. Additionally, IR is closely associated with a cluster of cardiometabolic abnormalities, including hypertension, hyperglycemia, dyslipidemia (especially elevated TG and low HDL-c), and visceral obesity, which are independent risk factors for ASCVD [32–34]. In this study, individuals in higher TyG quartiles (Q3 and Q4) exhibited significantly higher mean values of SBP, DBP, FBG, HBA1c, TG, VLDL-c, uric acid, WC and low HDL-c, which are hallmarks of metabolic syndrome. These findings suggest that the clustering of these metabolic disturbances may partly explain the elevated CVD risk observed in Q3 and Q4 [32,35].

Subgroup analysis revealed that higher quartiles of TyG index (Q3 and Q4) were associated with increased CVD risk in both males and females, with the effect being more pronounced in individuals aged ≥60 years. In the linear regression analysis only the female subjects had high odds of CVD. Several studies have reported conflicting sex differences in the association between TyG index and CVD. While some studies suggest that CVD risk may be higher in females with high TyG index, others indicate that both sexes experience elevated risk with increasing TyG index levels [27,36–38]. Increasing insulin resistance with post-menopausal state and its attendant reduced levels of estrogen might account for the higher CVD risk in females [37–39]. The mean age of our female subjects was 56.66 ± 13.9, an age range most females are likely to be post-menopausal. This might partially explain why CVD risk was higher in them. Additionally, age remains an immutable risk factor for CVD, with most cardiovascular risk factors becoming more prevalent with advancing age.

In the face of burgeoning burden of hypertension in Nigeria, its bi-directional relationship with IR and the attendant risk of CVD, the easy-to-measure TyG index is a reliable clinical tool to measure IR. Its ability to foretell CVD makes it doubly useful in the clinical assessment of hypertensives.

A notable strength of this study is that it is the first to examine the association between the TyG index and 10-year CVD risk in a Nigerian hypertensive population using FRS and also to determine cut-off points that reasonably predict 10-year risk of CVD. Additionally, the large sample size (1,430 participants) strengthens the reliability of the findings. However, it is cross-sectional in design and inherently unable to prove causality. Longitudinal studies are needed to establish dose-response relationships between the TyG index and incident CVD events. Furthermore, this study did not assess whether integrating the TyG index into existing cardiovascular risk prediction models improves their predictive power. Future research should investigate whether the incorporation of the TyG index enhances the accuracy of traditional risk stratification tools.

## Conclusion

The triglyceride-glucose (TyG) index was associated with an increased 10-year risk of cardiovascular disease in Nigerians with hypertension respecially in females, indicating its potential utility in cardiovascular risk assessment. However, further studies are needed to determine whether incorporating the TyG index into existing risk stratification models enhances predictive accuracy.

## Supporting information

S1 Data (XLSX)
